# Clinical efficacy of Curcuvet and Boswellic acid combined with conventional nutraceutical product: An aid to canine osteoarthritis

**DOI:** 10.1371/journal.pone.0252279

**Published:** 2021-05-28

**Authors:** Chiara Caterino, Federica Aragosa, Giovanni Della Valle, Dario Costanza, Francesco Lamagna, Alfonso Piscitelli, Annalisa Nieddu, Gerardo Fatone

**Affiliations:** 1 Department of Veterinary Medicine and Animal Production, University of Naples “Federico II”, Naples, Italy; 2 Interdepartmental Center of Veterinary Radiology, University of Naples “Federico II”, Naples, Italy; 3 Department of Agricultural Sciences, University of Naples “Federico II”, Portici, Italy; 4 Veterinary Division, Aurora Biofarma, Milano, Italy; University of Life Sciences in Lublin, POLAND

## Abstract

**Introduction:**

Osteoarthritis is a progressive degenerative joint disease which is high prevalent in dogs. In the late stage of the disease, it determines chronic neuropathic pain which leads to reduced quality-of-life in affected patients. To date it has not yet been identified a specific treatment, but it has been proved that nutraceutical and dietary supplements may play an important role in controlling inflammation and pain. The aim of this study was to evaluate, by the use of force plate gait analysis, the clinical efficacy of Boswellia and Curcuvet® combined with conventional nutraceutical therapy compared with conventional nutraceutical alone in dogs affected by osteoarthritis.

**Materials and methods:**

Twenty client-owned dogs, over 12 months old and 20 kg of body-weight, with a confirmed diagnosis of Osteoarthritis, were included in this randomized, double-blinded study. The dogs were randomly divided into two groups: the first group (A) received a conventional nutraceutical (consisted in a preparation of glucosamine, chondroitin sulfate, fish-oil containing 80% of omega 3-fatty acid, vitamin C and E, saccharomyces Cerevisiae) with a combination of acid boswellic and Curcuvet®, while the second group (B) received a conventional nutraceutical. All the enrolled dogs underwent a washout period before starting the treatment with nutraceuticals products which were the only admitted treatment over the study period. A full orthopaedic and neurologic examination, and force plate gait analysis were performed before starting the treatment, at 45, 90, and 60 days post-treatment. Ground reaction forces were recorded and analyzed.

**Results:**

Twenty dogs were enrolled in the study. In both groups there was an increasing values of ground reaction forces. These results might indicate that both nutraceutical products determined a better condition in terms of pain feeling but that effect is much more visible after 60 days from the end of the administration in treated group.

**Discussion:**

In conclusion Curcuvet in combination with Boswellic acid could be considered a valid aid in a multimodal treatment for canine osteoarthritis.

## Introduction

Osteoarthritis (OA) is a slowly progressive disease characterized by articular cartilage degradation, subchondral bone sclerosis, periarticular proliferation of new bone, and chronic inflammation of synovial membrane [1].

Aging alone is an unlikely cause of OA: in veterinary literature, it has been suggested that 20% of dogs above one year old are affected by the progressive changes of OA [2]. The OA should be considered a final common pathway of joint damage secondary to a multitude of inciting events, many of which may also be unrecognized [3, 4].

Clinical signs of OA are lameness, stiffness, loss of joint function, and mobility. In the late stage of the disease, chronic neuropathic pain leads to reduced quality-of-life of affected dogs [5, 6]. There is no specific OA treatment; multimodal management, including Non-steroidal anti-inflammatory drugs (NSAIDs) and nutraceuticals, is the preferred approach [7]. The NSAIDs effectively relieve the pain in canine OA, even if related long-term use side effects (e.g., duodenal ulcers, acute renal failure, increased enzyme activities) reduce the safety usage of these drugs [6].

Nutraceuticals are natural supplements widely used in veterinary medicine as a multimodal treatment of OA [8–13]. Glucosamine and chondroitin sulfate synergistically contribute to cartilage formation and repair [14]; they also decrease the signs of pain associated with OA [15]. In the last decades, several Authors reports the widespread use of plant derivatives as pharmaceutical grades nutrients [7]. In human medicine, the effectiveness of Curcuma and Boswellia extracts for OA treatment, inflammation, and wound healing is well known [9, 16]. Curcuma longa is an aromatic substance of vegetable origin with anti-inflammatory properties due to the capability to modulate several important molecular targets including pro-inflammatory enzymes (COX-LOX), transcription factor and cytokines [16] but a low bioavailability due to scares absorption and rapid metabolism, and systemic elimination [17].

A patented form of phytosomal turmeric for animal use with high bioavailability called Curcuvet® (Indena S.p.a., Milan, Italy) has been introduced as a nutraceutical adjuvant. Curcuvet® seems to cross the lipid-rich biomembranes and protect the valuable components of the turmeric extract from destruction by digestive secretions and gut bacteria [18]. Colitti in 2012 reported its valuable aid in the treatment of canine OA [19].

Boswellia serrata is a plant belonging to the Burseraceae family. The gummy resin extracted from Boswellia serrata is widely used in Ayurvedic medicine for its anti-inflammatory and anti-arthritic properties. Both human and veterinary literature has revealed the efficacy of these dietary supplements in OA treatment due to the prevention of collagen degradation and inhibition of pro-inflammatory mediators such as prostaglandis, COX, nitric oxide and NF-kB and down regulation of the pre-inflammatory cascade [7, 16]. Several authors reported the enhancement of OA management due to the combination of curcuma and boswellia. According to current literature, there are few data about the clinical efficacy of these nutraceuticals joined in a single trade product for manage canine OA [7].

The aim of this study was to evaluate the clinical efficacy of Boswellia and Curcuvet® combined with conventional nutraceutical therapy compared with conventional nutraceutical alone in dogs affected by OA.

## Material and methods

The randomized, double-blinded study was conducted at the Veterinary Teaching Hospital of University of Naples “Federico II”. Over 12 months old and 20 kg of body-weight, client-owned dogs with a confirmed diagnosis of OA were included in the study. Shoulder, elbow, and stifle joint were considered landmarks of OA secondary to joint disease and/or surgical treatment.

Two different products, no.2076 and no.2077, were prepared by Aurora Biofarma (Via Nicola Antonio Porpora 127, 20131, Milan, Italy) and presented as tablets of the identical appearance contained in two same no-marked boxes. The two nutraceutical drugs consisted in a preparation of glucosamine (GS), chondroitin sulfate, fish-oil (containing 80% of omega 3-fatty acid), vitamin C and E, saccharomyces Cerevisiae. In one of them, boswellic acid and Curcuvet® were added. Product no.2076 and no.2077 were randomly assigned to groups A or B, using online randomization (https://www.randomizer.org). Only after data analysis ended, the Authors knew the treatment code providing the information about the actual assignments of group A–which received the product with Boswellic Acid and Curcuvet® (no.2076)–and group B–which received the product without them (no.2077).

A two-week withdrawal period was required for NSAIDs and short-acting glucocorticoids and one-month for long-acting oral or parental glucocorticoids.

Anti-inflammatory medications (e.g., NSAIDs or glucocorticoids) during the study period, concomitant neurological or systemic disease with an inflammatory component represent exclusion criteria. The dogs were allowed to carry out their normal physical activity, but no physiotherapy.

A full orthopaedic and neurologic examination and force plate gait analysis were performed before starting the treatment (T0), at 45 (T1) and 90 days (T2) of treatment, and at 60 days (T3) post-treatment for an overall study period of 5 months. Clinical evaluations were performed by the same investigators (GDV, CC). Body-weight (BW), presence/absence of a palpable joint effusion, pain during manipulation were recorded at each time point. The lameness was objectively assessed using a computer-assisted force platform gait analysis (PASCO Capstone software version 2.2.2). At T0 and T1, each owner received the nutraceutical drugs, daily administered at dose of 1 tablet/10 kg of body-weight, to guarantee 90 days of treatment followed by 60 days of discontinuation of nutraceutical.

### OA score

The OA score was assessed according to Morgan *et al*. in 2010 for the stifle [20]; according to International Elbow Working Group (IEWG) for the elbow joint and graded in four groups (1 = no OA; 2 = mild OA; 3 = moderate OA; 4 = severe OA) for the shoulder [20].

All dogs were, then, categorized into grades 0–3 according to the following classification: 0 = no OA, 1 = mild OA, 2 = moderate OA, 3 = severe OA [20].

### Forces gait plate analysis

Force plate gait analysis was performed at T0, T1, T2 and T3. A 40x40 cm platform (PASPORT Force Platform, PS-2141, PASCO scientific, California, USA) placed in a 4m walkway was used to record GFRs.

Before data collection, dogs were let walking free across the walkway for at least 15 minutes to familiarize with the environment and the operators. Each trial was considered valid when the pelvic limb and the thoracic limb fully struck at the same time the surface of the plate. The dogs were walked over the pressure plate until five valid trials were achieved. The dog’s velocity was registered with a dedicated detector (Motion Sensor II, CI-6742, PASCO scientific, California, USA) and only trials with a velocity of 1–1.3 m/s were accepted. Dogs were walked in both directions with a standardized starting position [21].

Force-to-time curve was generated by the computer-analysis system (PASCO Capstone™ software 2.2.2, PASCO scientific, California, USA). Registered kinetic GFRs were collected for both pelvic limbs and included peak of vertical force (PVF) and vertical impulse (VI). PVF was defined as the maximum force exerted perpendicular to the surface during the stance phase, while VI was the calculated area under vertical force curve during time. As previously described, the GRFs parameters were normalised to body weight (PVF%BW, VI%BW) [22].

## Statistical analysis

The collected data were analyzed using a specific statistics software package (IBM® SPSS® Statistics Version 26.0, IBM Corporation, Armonk, New York). After verifying that the data of normalized kinetic variables (PVF%BW_T0_, PVF%BW_T1_, PVF%BW_T2_, PVF%BW_T3_, VI%BW_T0_, VI%BW_T1_, VI%BW_T2_ and VI%BW_T3_, ST_T0_, ST_T1_, ST_T2_, ST_T3_) were not normally distributed through Kolmogorov–Smirnov test, a Mann–Whitney U-test was used to examine, at time T_0_, the differences between groups A and B, in order to check whether randomization had divided the dogs into two homogeneous groups, in terms of PVF%BW, VI%BW, ST, and body weight (W).

In order to check whether each of the two nutraceutical therapies may have had an effect on OA at the end of the observation time, a 2-tailed Wilcoxon matched-pairs signed rank test was used to examine the differences between pre- and post-treatment (baseline and at 150 days) values on lame limbs for each group.

Finally, in order to compare differences within each nutraceutical therapy over different time points, Friedman’s ANOVA test for related samples was used to measure the significance of the increase in the GRFs from T_0_ to T_3_ for each sick limb for both groups. Pair-wise multiple comparisons, provided by Dunn-Bonferroni test, were used as a post hoc test.

The significance level for all statistical tests was set a priori at p≤0.05.

## Results

Twenty dogs met the inclusion criteria: 11 were male (1 neutered), 9 were female (5 neutered). Table 1 shows the distribution of canine breeds. Mean ± standard deviation age in months was 58 ±34.1. Nine dogs had a history of elbow dysplasia and signs of OA after surgical treatment for fragmented coronoid process (FCP); eleven dogs had a history of cranial cruciate ligament failure and signs of OA after Modified Maquet procedure. No one dog show shoulder OA.

**Table 1:** Distribution of study population for breed and sex.

Breed	Group		Total
	A	B	
**Labrador Retriever**	3 F+3 M	3 M	9
**American Pitbull Terrier**	.	1 F	1
**Rottweiler**	1 M	1 F	2
**Italian Spinone**	.	1 M	1
**Cross breed**	4 F	3 M	7
**TOTAL**	**11**	**9**	**20**

### The dogs were randomly divided into two groups: Treatment (A) and control (B)

In the A group, 5 dogs had elbow joint OA, 5 stifle OA; in the B group, 4 dogs had elbow joint OA, and 6 at stifle OA. The mean BW was 35.04 ± 4.89 kg and 35.36 ± 4.66 kg for group A and B, respectively. Mean ± standard deviation OA grade in group A was 2.3 ± 0.48, while in group B was 2.2 ± 0.63. At T0, no statistical difference for GFRs, and BW between the two groups was present, meaning that at T0, the groups can be considered homogeneous (see Table 2).

**Table 2:** Body-weight and GFRs at time T_0_: Ranks and Mann–Whitney U-test values.

Variables	Group	N	Mean rank	Sum of ranks	U Mann-Whitney	*p-value*
W_T_0_	A	10	10.3	103.00	48.0	0.879
	B	10	10.7	107.00		
PVF%BW_T_0_	A	10	8.9	89.00	34.0	0.226
	B	10	12.1	121.00		
VI%BW_T_0_	A	10	9.2	92.00	37.0	0.326
	B	10	11.8	118.00		
ST_T_0_	A	10	11.35	113.50	41.5	0.519
	B	10	9.65	96.50		

In group A the statistical comparison between T0 and T3 showed a statistically significant increase in VI%BW (*p-value* = 0.009) and ST (*p-value* = 0.021), while, although 8/10 cases showed a PVF%BW value at T3 higher than T0, the increase is not statistically significant (see Table 3).

**Table 3:** GRFs: Ranks and Wilcoxon signed rank test values–group A: Product 2076.

Variable	Types		N	Mean Rank	Sum of Ranks	Wilcoxon Signed Ranks Test (based on negative ranks)	*p-value*
**PVF%BW**	Negative Ranks	(Post<Pre)	2	6.00	12.00	-1.580	0.109
	Positive Ranks	(Post>Pre)	8	5.38	43.00		
	Ties		0				
**VI%BW**	Negative Ranks	(Post<Pre)	1	2.00	2.00	-2.599	0.009
	Positive Ranks	(Post>Pre)	9	5.89	53.00		
	Ties		0				
**ST**	Negative Ranks	(Post<Pre)	1	5.00	5.00	-2.293	0.021
	Positive Ranks	(Post>Pre)	9	5.56	50.00		
	Ties		0				

On the other hand, for group B, statistical comparison between T0 and T3 showed a statistically significant increase only in VI%BW (*p-value* = 0.028), while in the cases of PVF%BW and ST there was not a statistically significant increase (see Table 4).

**Table 4:** GFRs: Ranks and Wilcoxon signed rank test values–group B: Product 2077.

Variable	Types		N	Mean Rank	Sum of Ranks	Wilcoxon Signed Ranks Test (based on negative ranks)	*p-value*
**PVF%BW**	Negative Ranks	(Post<Pre)	2	4.50	9.00	-1.886	0.059
	Positive Ranks	(Post>Pre)	8	5.75	46.00		
	Ties		0				
**VI%BW**	Negative Ranks	(Post<Pre)	2	3.00	6.00	-2.191	0.028
	Positive Ranks	(Post>Pre)	8	6.13	49.00		
	Ties		0				
**ST**	Negative Ranks	(Post<Pre)	4	3.00	12.00	-1.245	0.213
	Positive Ranks	(Post>Pre)	5	6.60	33.00		
	Ties		1				

Moreover, in order to determine the significance of the increase in the values of GFRs measured on the affected limb, from T_0_ to T_3_, the Friedman’s ANOVA test for related samples was performed on each group. The increase of VI%BW across the overall time of the study does not differ significantly in group B. On the contrary, in group A the increase of VI%BW is statistically significant overall the study period. Despite the overall increase in the study period, PVF%BW and ST values do not differ significantly in either group A or group B (see Table 5).

**Table 5:** GFRs: Quartiles for each time points and Friedmans’ ANOVA p-values.

	**PVF%BW**								
	T_0_		T_1_		T_2_		T_3_		
	**Median**	**Q1-Q3**	**Median**	**Q1-Q3**	**Median**	**Q1-Q3**	**Median**	**Q1-Q3**	***p-value***
**Group A**	48.21	[29.74–57.78]	44.95	[36.43–56.25]	46.09	[34.75–59.09]	47.24	[35.91–59.64]	0.112
**Group B**	30.13	[28.90–46.33]	41.08	[33.73–59.33]	37.67	[30.30–60.45]	38.22	[32.93–59.25]	0.052
	**VI%BW**								
	T_0_		T_1_		T_2_		T_3_		
	**Median**	**Q1-Q3**	**Median**	**Q1-Q3**	**Median**	**Q1-Q3**	**Median**	**Q1-Q3**	***p-value***
**Group A**	18	[13.46–23.54]	20.40	[14.73–24.52]	19.64	[15.10–24.56]	22.38	[16.72–27.32]	0.001
**Group B**	15.5	[11.93–20.61]	17.98	[15.39–24.57]	16.7	[14.13–23.38]	19.35	[16.20–25.43]	0.116
	**ST**								
	T_0_		T_1_		T_2_		T_3_		
	**Median**	**Q1-Q3**	**Median**	**Q1-Q3**	**Median**	**Q1-Q3**	**Median**	**Q1-Q3**	***p-value***
**Group A**	0.6	[0.557–0.717]	0.661	[0.62–1.0]	0.667	[0.579–1.0]	1	[0.665–1.0]	0.071
**Group B**	0.685	[0.525–1.0]	0.711	[0.565–1.0]	0.642	[0.553–0.691]	1	[0.65–1.0]	0.071

## Discussion

This randomized, double-blind trial shows the usefulness of curcuvet® and boswellic acid to reduce the lameness and pain in dogs with OA. All dogs recruited were free of any compound purported to relieve the clinical signs of OA in order to provide rigorous evidence of the therapeutic potential of curcuvet® and boswellic acid.

Force-plate analysis has been used in several studies as a method to objectively compare the clinical outcome of different surgical techniques or medications [23, 24]. Use of force-plate analysis provided accurate and repeatable data on limb function and objective measurement of the efficacy of nutraceutical treatment [23].

**Fig 1:**
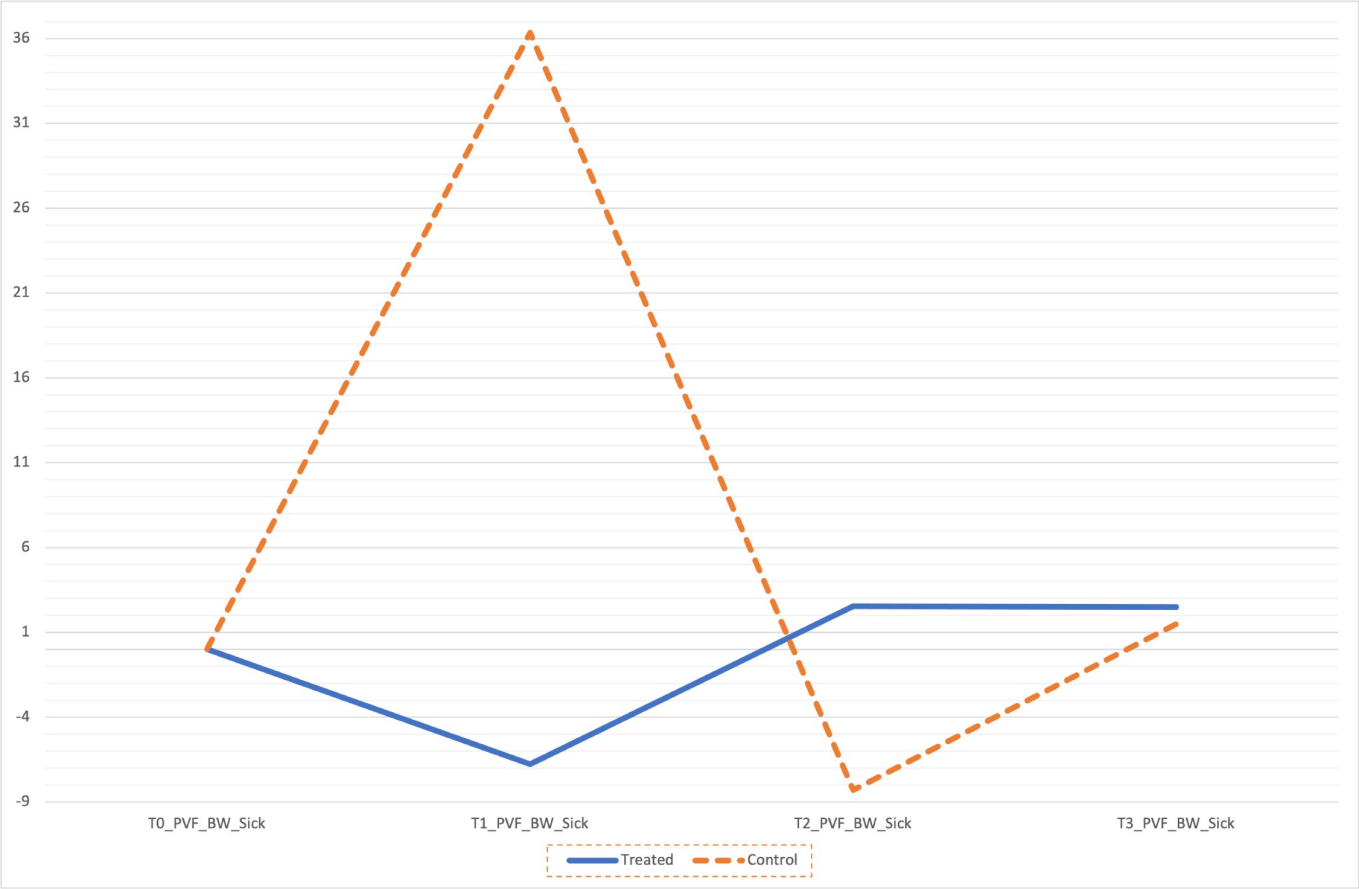
Percentage increase in PVF%BW over time between two groups.

Comparing gait analysis data of thoracic and pelvic limbs in lame dogs could make difficult the data interpretation since forelimb PVF is normally higher than hind limbs [25]. Our data analysis at T0 show the homogeneity of two groups for OA localization, score, GFRs and BW avoiding rescaling, harmonization, or normalization of them which are necessary steps in comparative studies [26]. Peak Vertical Force and VI are considered suitable index evaluating limb function; in particular, PVF is defined as the maximum force exerted perpendicular to the surface during stance phase (ST); while VI is the calculated area under vertical force curve using time. Therefore, in lameness dog, a decreased PVF denotes a less bear weight and a resulting reduced ST and VI. In this clinical trial, we experienced an improvement of PVF in 16/20 (80%) of patients, despite a no statistical significance, in both groups showing as the nutraceutical can ameliorate the pain perception and therefore lameness. Moreover, the dogs receiving curcuvet® and boswellic acid, showed a higher and constant overall time PVF%BW mean values until the end point of study, than control group (Fig 1) [6, 27].

**Fig 2:**
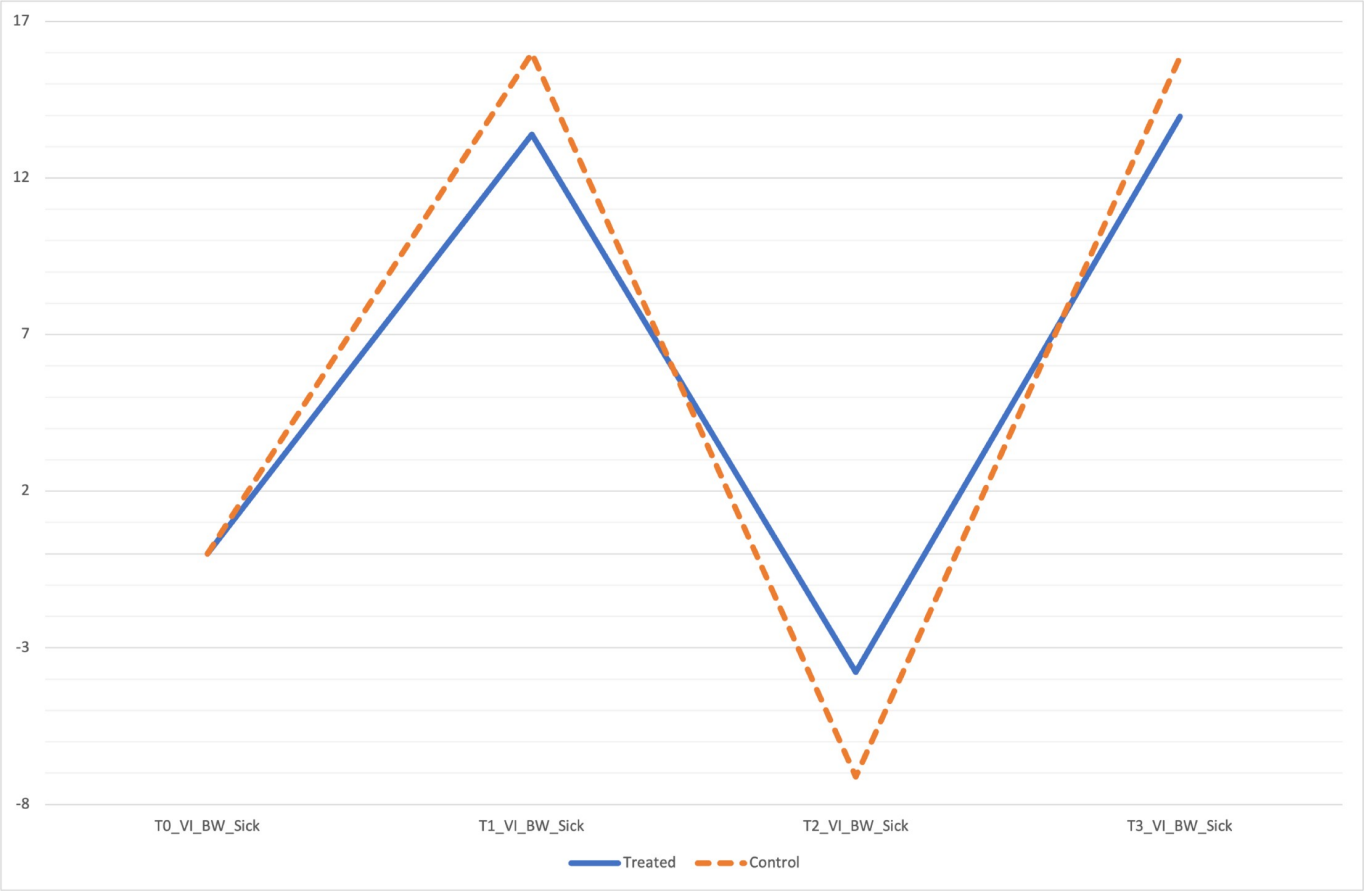
Percentage increase in VI%BW over time between two groups.

**Fig 3:**
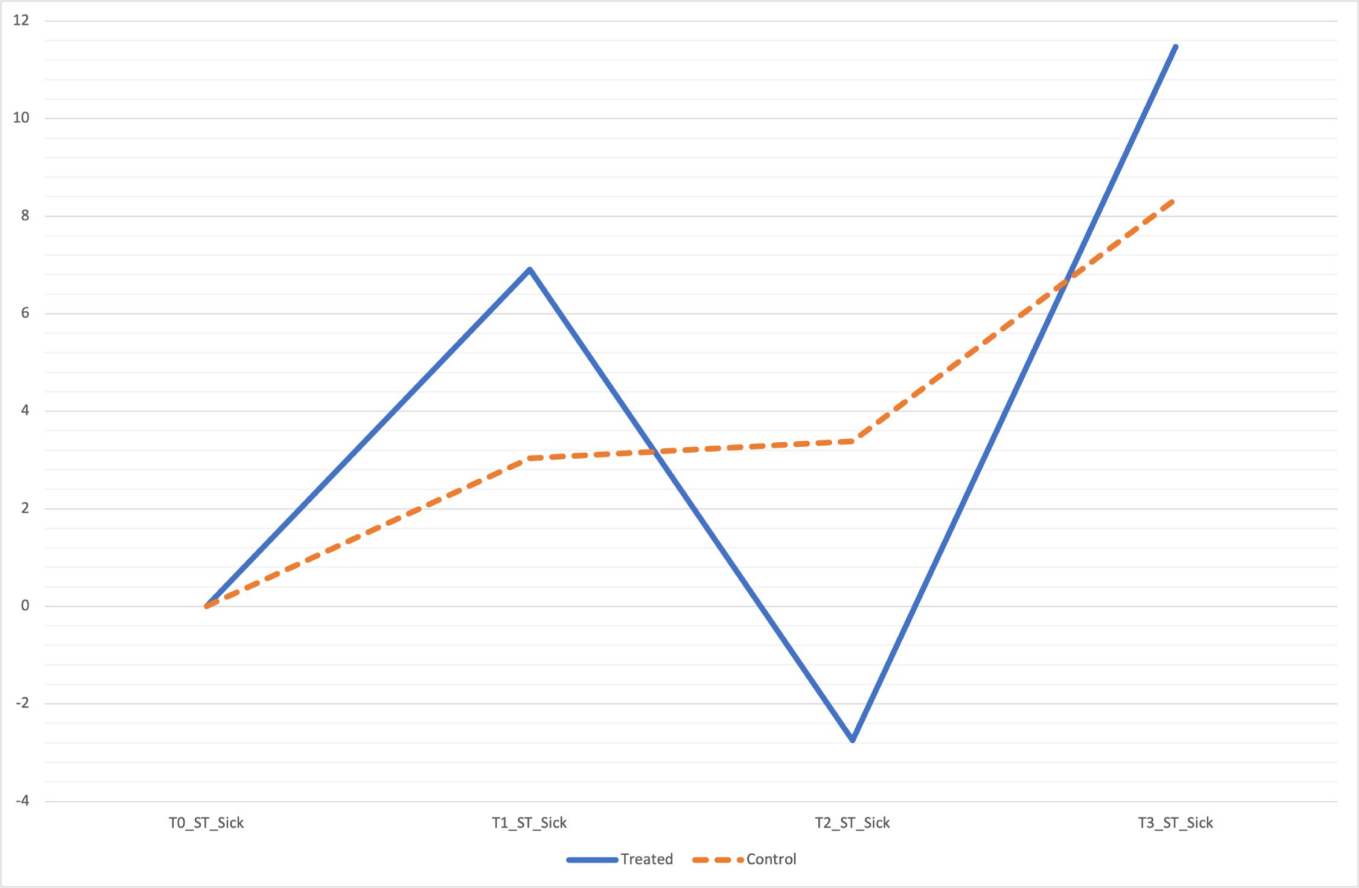
Percentage increase in ST over time between two groups.

Besides, a statistically significant improvement of VI%BW and ST in treated dogs across overall time detect an amelioration of limb function, underlining the therapeutic power of curcuvet® and boswellic acid (Figs 2 and 3) [28]. Other components of the force curve are rising slope (RS) and falling slope (FS), defined as the period from baseline value at ground initial contact to the maximal force and the period from maximal force to when contact with the ground ends, respectively. In lame dogs the RS is reduced and the FS is increased due to the cautious initial bear load and a quicker removal of weight from the limb, respectively. In this clinical trial we observed that the RS was steeper in treated dogs meaning more rapid bear loading whilst the FS less steep showed a slower offloading of weight.

Since curcumin and boswellic acid are chemically quite different, consequently their targets are also likely different, so a combination of them could explain their synergistic action, as reported in human and veterinary literature [29]. The clinical relevance of our results suggests that curcuma and boswellic acid have played a key role in inflammation and pain relief and, also, is consistent with literature [30].

The OA is a chronic degenerative condition, and no treatment can stop it. The most common treatment is based on a multimodal approach, combining NAISDs and nutraceuticals. Although, in veterinary practice, NSAIDs’ administration is limited by the side effects on long-term use [31]. In this clinical trial we treated dogs with a mild degree of OA only with nutraceuticals administration for 90 days with objectively satisfactory results, yet visible after 60 days from the end of the treatment.

In our opinion the anti-inflammatory property of curcuvet® and boswellic acid, may mitigate these side effects, reducing the dose to the lowest daily effective.

In conclusion the combination of boswellic acid and curcuvet® seems to be a valid support in the treatment of dogs with OA.
